# High Intensity Aerobic exercise training and Immune cell Mobilization in patients with lung cancer (HI AIM)—a randomized controlled trial

**DOI:** 10.1186/s12885-022-09349-y

**Published:** 2022-03-05

**Authors:** Gitte Holmen Olofsson, Marta Kramer Mikkelsen, Anne-Mette Ragle, Anne Birgitte Christiansen, Anne Pries Olsen, Lise Heide-Ottosen, Cecilia Bech Horsted, Cia Moon Scharbau Pedersen, Lotte Engell-Noerregaard, Torben Lorentzen, Gitte Fredberg Persson, Anders Vinther, Dorte Lisbet Nielsen, Per thor Straten

**Affiliations:** 1 National Center for Cancer Immune Therapy, CCIT-DK, Department of Oncology, Copenhagen University Hospital Herlev and Gentofte, Herlev, Denmark; 2 Department of Oncology, Copenhagen University Hospital Herlev and Gentofte, Herlev, Denmark; 3 Department of Physiotherapy and Occupational Therapy, Herlev and Gentofte Hospital, Herlev, Denmark; 4 Department of Gastric Surgery, Ultrasound Section, Herlev and Gentofte Hospital, Herlev, Denmark; 5 Faculty of Health and Medical Sciences, University of Copenhagen, Copenhagen, Denmark; 6 Hospital Secretariat and Communications, Research, Herlev and Gentofte Hospital, Herlev, Denmark; 7 Department of Immunology and Microbiology, Faculty of Health and Medical Sciences, University of Copenhagen, Copenhagen, Denmark

**Keywords:** Aerobic exercise, Exercise, Physical activity, Lung cancer, Cancer, Immunotherapy, T cells, NK cells

## Abstract

**Background:**

The increasing role of exercise training in cancer care is built on evidence that exercise can reduce side effects of treatment, improve physical functioning and quality of life. We and others have shown in mouse tumor models, that exercise leads to an adrenalin-mediated increased influx of T and NK cells into the tumor, altering the tumor microenvironment (TME) and leading to reduced tumor growth. These data suggest that exercise could improve immune responses against cancer cells by increase immune cell infiltration to the tumor and potentially having an impact on disease progression. Additionally, there are data to suggest that infiltration of T and NK cells into the TME is correlates with response to immune checkpoint inhibitors in patients. We have therefore initiated the clinical trial HI AIM, to investigate if high intensity exercise can mobilize and increase infiltration of immune cells in the TME in patients with lung cancer.

**Methods:**

HI AIM (NCT04263467) is a randomized controlled trial (70 patients, 1:1) for patients with non-small cell lung cancer. Patients in the treatment arm, receive an exercise-intervention consisting of supervised and group-based exercise training, comprising primarily intermediate to high intensity interval training three times per week over 6 weeks. All patients will also receive standard oncological treatments; checkpoint inhibitors, checkpoint inhibitors combined with chemotherapy or oncological surveillance. Blood samples and biopsies (ultrasound guided), harvested before, during and after the 6-week training program, will form basis for immunological measurements of an array of immune cells and markers. Primary outcome is circulating NK cells. Secondary outcome is other circulating immune cells, infiltration of immune cells in tumor, inflammatory markers, aerobic capacity measured by VO_2_ max test, physical activity levels and quality of life measured by questionnaires, and clinical outcomes.

**Discussion:**

To our knowledge, HI AIM is the first project to combine supervised and monitored exercise in patients with lung cancer, with rigorous analyses of immune and cancer cell markers over the course of the trial. Data from the trial can potentially support exercise as a tool to mobilize cells of the immune system, which in turn could potentiate the effect of immunotherapy.

**Trial registration:**

The study was prospectively registered at ClinicalTrials.gov on February 10^th^ 2020, ID: NCT04263467. https://clinicaltrials.gov/ct2/show/NCT04263467

## Background

Lung cancer (LC) is the second most common cancer and the leading cause of cancer-related deaths with 2.2 million new cases and 1.9 million deaths worldwide in 2020 [1]. Non small-cell lung cancer (NSCLC) accounts for about 85% of LC cases and can be divided into squamous cell- and non-squamous carcinomas [2]. Non-squamous carcinomas accounts for nearly 70% of NSCLC and the vast majority are adenocarcinomas [1]. The prognosis is poor, as most patients have advanced or metastatic disease at diagnosis and a large proportion of patients, that are treated curatively, relapse subsequently.

Immunotherapy has revolutionized cancer treatment within the last decade [3] and was approved for second line treatment of NSCLC in 2015 [4, 5]. The Danish Cancer Registry reports of improvement in the overall survival rates from the 2009–2013 to the 2014–2018 time period. The 1-year survival have improved from 40–48% and 47–56%, and the 5-year survival rate from 14–20% and 20–27% in men and women respectively (NORDCAN-IARC). Immune checkpoint inhibitors (CPI) are monoclonal antibodies that targets the immune system and/or tumour cells, which releases T cells from an inhibitory receptor-ligand interaction typically between T cells and other cells of the immune system, or T cells and cancer cells. The breach of inhibitory signalling in the T cell allow more efficient anti-cancer immune responses [6]. CPI targeting the programmed cell death protein 1 (PD-1), its ligand (PD-L1) or the cytotoxic T lymphocyte associated protein 4 (CTLA-4). Major phase III clinical trials with CPIs have generated extensive data and changed first line treatment regimens to include immunotherapy for patients with NSCLC. These trials have focused on CPI monotherapy, CPI double blockade or CPI in combination with chemotherapy [7–16]. The impressive clinical results have highlighted the potential role of the immune system in NSCLC.

A major challenge, however, is the lack of strong predictive markers for response to immunotherapy. Although PD-L1 expression in the TME and/or the tumor cells is being used for some indications, PD-L1 expression in most cases inadequately selects patients that are prone to response [17]. Other markers under scrutiny are tumor mutation burden (TMB) and tumor infiltrating lymphocytes (TIL). Both high level of TMB and TIL have been shown to correlate with response to CPI therapy in several small exploratory studies [18].

Exercise training is a strengthening multi-effect strategy with capacity to work across multiple organ systems. Numerous intervention studies have shown that exercise training has beneficial effects in patients with cancer [19]. Documented effects include reduction of symptoms and side effects, improved physical capacity and functioning, increased muscular strength, and improvements in quality of life (QoL) [20–25]. These improvements have been demonstrated both during and after anti-cancer treatment, and across several cancers, disease stages and treatment regimens [19–23]. There is also growing epidemiological evidence that a physical active lifestyle is associated with lower risk of several cancers [26], most clearly elucidated in colon [27], breast [28] and endometrial cancer [29]. In addition, some observational studies have shown that a physical active lifestyle is associated with reduced risk of cancer recurrence and mortality in cancer survivors [30].

Studies in mouse tumor models have shown that exercise-mediated changes in hormone levels, inflammation, and immune cell function may play central role in modulation of the TME [31]. We recently found that voluntary exercise reduced tumor growth in several mouse models [32]. Tumors from exercising mice were infiltrated to a much greater extent by immune cells (NK and T cells). We could show that the mobilization of the immune cells was adrenaline-dependent, and that this mobilization subsequently increased influx of immune cells to the tumor. Since elevated numbers of CD8 T cells may increase the chance for response to treatment with CPI, we suggest that exercise could constitute a simple (and healthy) way by which the response rate for patients on immunotherapy could be increased [33]. To test these hypotheses, we have initiated the randomized controlled trial **H**igh **I**ntensity **A**erobic exercise training and **I**mmune cell **M**obilization in patients with lung cancer (**HI AIM**).

## Methods

### Design and setting of the study

HI AIM is a prospective two-armed randomized controlled trial. The trial is led by departments across Herlev and Gentofte Hospital, including National Center for Cancer Immune Therapy (CCIT-DK); Department of Oncology; Department of Physiotherapy and Occupational Therapy; and Department of Gastric Surgery, the Ultrasound Section. The study is prospectively registered at clinicaltrails.gov (NCT04263467). Ethics approval has been obtained from the Ethics Committee at Capital Region (H-19031814). Finally, the Danish Data Protection Agency has approved the project (P-2019–278). This article describes the design of the study according to SPIRIT guidelines for RCT.

### Patients

A total of 70 patients with metastatic NSCLC will be recruited from Department of Oncology, at Herlev and Gentofte Hospital. Screening for eligible patients will be carried out by the oncologists in the department. Patients are required to sign an informed consent prior to inclusion and the research team will obtain ICF and all relevant baseline data before randomization. These will be stored according to danish legacy.

Patients are eligible for inclusion if the following inclusion criteria are fulfilled: 1) Diagnosed with metastatic NSCLC, 2) Measurable disease according to RECIST 1.1, 3) Age ≥ 18 years, 4) Treatment with CPIs, CPIs combined with chemotherapy or oncological surveillance, 5) Eastern Cooperative Oncology Group (ECOG) performance status score (PS) ≤ 2, 6) Preferable metastasis suitable for biopsy, 7) Normal bone marrow function, and, 8) Willingness to give informed consent for participation in the study.

Exclusion criteria are: 1) Any physical condition that hinder the execution of physical exercise, 2) Severe dyspnea that hinder the execution of high intensity aerobic exercise training, 3) Symptomatic brain metastases, 4) Dementia, psychotic disorders, or other cognitive diseases or conditions that hinder participation in a clinical exercise-based trial, 5) Unstable medical disease or history of serious or concurrent illness; any medical condition that might be aggravated by exercise training or that cannot be controlled, including, but not restricted to congestive heart failure (NYHA class III-IV), unstable angina pectoris, implantable cardioverter defibrillator (ICD), or myocardial infarction within 6 months, 6) A condition requiring systemic treatment with either corticosteroids (> 10 mg daily prednisone equivalents) or other immunosuppressive medications. Inhaled or topical steroids and adrenal replacement doses ≤ 10 mg daily prednisone equivalents are permitted. 7) Use of beta blockers, 8) Any systemic infections within the last 4 weeks, 9) Bone metastases, where exercise training increases the risk of pathological fracture as assessed by the treating physician.

### Randomization

After inclusion, participants are randomly allocated to the intervention group or control group, using a 1:1 block randomization. ‘Blockrand’ package of the statistical software R was used to generate all randomization lists, and block size will only by known by the statistician setting it up. Randomization will be administrated via the web-based research platform REDCap (Research Electronic Data Capture). Randomization will be stratified according to treatment being: Immunotherapy, immunotherapy combined with chemotherapy or oncological surveillance. Source codes to generate all randomization lists are stored to ensure full reproducibility.

### Intervention

**Fig. 1:**
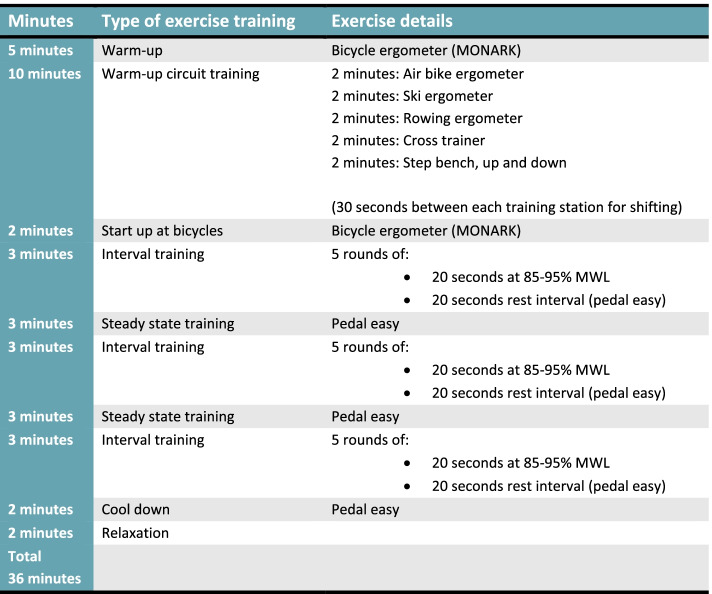
Illustration of one training session in the HI AIM protocol. MWL = maximal workload

Patients in the intervention group will receive an exercise-based intervention, in combination with standard treatment being immunotherapy, immunotherapy combined with chemotherapy or oncological surveillance. The exercise intervention consists of supervised and team-based (maximal team size: 5 participants) aerobic exercise training, three times a week for six weeks. Each training session will take approximately one hour, with effective time of training being ~ 40 min. Each training session will be initiated with 5 min of warm-up on a bicycle ergometer (Motion cycle 200 med, Emotion Fitnes GmbH & CO, Hochspeyer, Germany), followed by 5 × 2 min warm-up circuit training on: 1) an air bike ergometer (ReNegaDE AIR BIKE C2, Taiwan), 2) a ski ergometer (CONCEPT 2 Deutschland GmbH, Hamburg, Germany), 3) a rowing ergometer (Hiit Console, Core Health & Fitness LLC, Vancouver, USA), 4) a cross trainer (motion cross 600, Emotion Fitnes GmbH & CO, Hochspeyer, Germany), and, 5) exercises on a step bench. There will be 30 s between each exercise station for shifting. If a patient is unable to perform one of the exercises in the warm-up circuit training, it will be replaced by the bicycle ergometer. After the circuit training, the participants will shift to the bicycle ergometers for the rest of the session. The bicycle training will be initiated with 2 min start-up bicycling, followed by 3 × 3-min sequences of high intensity interval training constantly shifting between: A) 20 s at 85–95% MWL (as hard as you can go for 20 s) and B) 20 s rest interval (pedal easy). The high intensity interval sequences will be separated by two steady state sequences, each lasting 3 min. The training session will be finished off with 2 min of cool down cycling (pedal easy) and 2 min of stretching. Finally, a short session of mental relaxation will round up the training session for the participants. An overview of a training session is illustrated in Fig. 1.

Entering the study, MWL will be measured using a VO_2_ max test (described beneath). MWL will provide the basis for individualized training intensities in the interval training, furthermore, the physioterapist will check patients adherence to 85–90% MWL during the training sessions. A nurse will be present at all training sessions ensuring the well being of the patients. Adherence and any adverse events will be reported at each training session.

### Control

Patients in the control group will receive standard treatment being immunotherapy, immunotherapy combined with chemotherapy or oncological surveillance. No restrictions on physical activity are made.

### Study endpoints

The primary endpoint is to investigate the effect of exercise on circulating NK cells in patients with NSCLC, comparing the control and intervention group. Secondary endpoints include feasibility measures, the effect of exercise on circulating T-, B- and myeloid cells, changes in circulating biomarkers of inflammation, changes in immune cell infiltration in tumor, patient’s maximal aerobic capacity (VO_2_ max) and clinical effect in the form of overall response rate (ORR), progression free survival (PFS) and overall survival (OS). The outcome measurements are described in the following sections.

### Study assessment

**Fig. 2:**
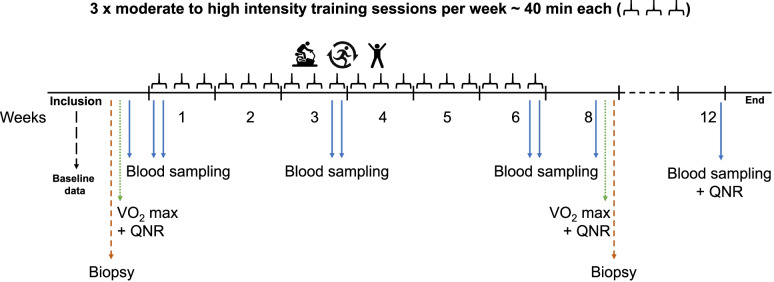
Overview of HI AIM study intervention. Shown here is the timeframe of the exercise-intervention and at which timepoints baseline data, questionnaires (QNR), VO_2_ max tests, tumor biopsies and blood samples are collected

Monitoring and assessment of the HI AIM trial consist of baseline information, questionnaires, VO_2_ max test, blood samples, biopsies and clinical data. All tests, data and samples will be collected from both the control – and interventions group. Monitoring of the trial is built around the six-week exercise intervention. Thus, samples, test and data will primarily be collected at baseline, after 8 weeks and at 12 weeks. The setup is described in detail in the following section, and Fig. 2 provides an overview of the timeframe.

#### Baseline data

Demographic and patient-reported data on civil status, working status, education level, smoking history and alcohol consumption will be collected. These data will only be collected at baseline.

#### Questionnaires

HI AIM will include three questionnaires, focusing on physical activity and mental health. Level of physical activity will be measured by self-report using the short version of the International Physical Activity Questionnaire (IPAQ) [34]. This questionnaire was originally developed as an instrument for cross sectional assessment of physical activity and has been widely used and validated internationally. The IPAQ has acceptable validity and reliability in the assessment of physical activity among adults aged 18–65 [34]. To cater for inclusion of participants in different age groups, level of physical activity will also be assessed using the Saltin and Grimby scale [35], in a modified version by Schnohr et al*.* [36]. This version has also been used in the Copenhagen City Heart Study – and is therefore known as ‘The Copenhagen City Heart Study Leisure Time Physical Activity Questionnaire’. Levels of anxiety and depression will be assessed using Hospital Anxiety and Depression Scale (HADS) [37]. Questionnaires will be sent out electronically at baseline, after 8 and 12 weeks. All data from the questionnaires will be stored in the electronic database REDCap.

#### *VO*_*2*_* max test*

Maximal aerobic capacity (VO_2_ peak/max) will be measured during an incremental exercise test conducted on a bicycle ergometer (CPET Vyntus CPX, Ergometer ViaSprint 150p, Vyarie Medical GmbH, Höchberg, Germany). VO_2_ max represents the maximal rate at which oxygen is transported to and utilized by the muscles [38], meaning the point where oxygen uptake no longer increases with increasing workload. At VO_2_ max, the participants’ maximal heart rate (HR_peak_) and MWL will also be measured. This will allow us to assess potential changes in aerobic capacity from pre to post intervention in both groups (measured at baseline and in week 7–8). All VO_2_ max test will be carried out by physiotherapist who is blinded to patient’s group randomization. These will maintain blinded until finalization of the trial.

#### Blood samples

Blood samples will be taken to measure immunological responses, including circulating immune cells and inflammatory biomarkers. For the control group, blood samples will be taken at baseline, week 4, week 7–8 and in week 11–12. For the intervention group, blood samples will be taken at baseline, week 7–8 and in week 11–12. Additionally, the intervention group will also have blood samples taken before and immediately after three training sessions (see Fig. 2): the first training session (week 1), 9^th^ session (week 3) and the last exercise session (week 6). Blood samples will be stored as serum, plasma and cryopreserved blood cells (in ampules of max 30 × 10^6^ cells) in CCIT’s biobank, Department of Oncology, Herlev and Gentofte Hospital until analyses.

#### Biopsies

Biopsies will be taken to evaluate several parameters of the TME. Efforts will be made to take biopsies from available tumor lesions at baseline and at week 8. Patients with NSCLC will primarily have tumor lesions in lung, liver, adrenal gland, bone or brain, and biopsies will only be taken from tumor lesions that are easily assessable. This will be evaluated by the responsible physician. A specialized physician at the radiology department will conduct the biopsy under sterile conditions via ultrasound guidance with a medium sized needle (biopsy size: 1.2 * 20 mm). Between 1–3 samples will be taken from each tumor lesions. Tumor samples will be stored in CCIT’s biobank, Department of Oncology, Herlev and Gentofte Hospital until analyses.

#### Clinical data

Clinical data, in the form of time of diagnosis, tumor stage, treatment regimen, ECOG PS, and comorbidities as assessed by Charlson comorbidity index (CCI) [39], weight, height, body mass index (BMI), hematology/biochemistry, and medication will be registered and assessed from medical records. CT scan of the thorax and abdomen is required at baseline (within 28 days before inclusion). Tumor response will be evaluated at baseline and at following CT scans (every 3 months according to the standard) and according to the RECIST 1.1 and iRECIST. To investigate whether the intervention may affect PFS and OS, recurrence and death (cancer-related and other causes) will be registered throughout the intervention period and during follow-up from medical records.

#### Feasibility and safety

Feasibility of the intervention will be evaluated as attrition and adherence. Attrition is the number of patients who do not complete the study (dropouts). Adherence to the intervention will be assessed as the number and percentage of attended training sessions. An adherence rate of 70% is set as criteria for fulfilling the exercise program. Safety (adverse events) and tolerability will be evaluated at every training session and documented within the electronic case report form in REDCap. Finally, safety and adverse events will be documented according to Ethics Committee at Capital Region requirements.

#### Analyses

Baseline characteristics and questionnaires will be presented and calculated for the intervention -, control and the whole group. Data will be collected in the database in electronic database REDCap. All feasibility measurements will be reported as numbers and percentages.

To assess the effect of exercise on the immune system in patients with NSCLC, key focus is on the composition of mobilized immune cells in peripheral blood, phenotypic changes of immune cells, changes in serum markers and infiltration of immune cells in tumor. Main studies on blood samples include flow cytometry analyses to study which cell types are mobilized, therefore, whole blood from all blood samples will be analyzed using a standard kit from BD Bioscience: 6-color TBNK Reagent with BD Truecount Tubes (Catalog No. 337166). Furthermore, flow cytometry (and potentially CYTOF) is used to measure frequencies and phenotypic changes of circulating NK cells, T cells, B cells, and MDSC within peripheral blood in response to exercise. Elisa and Luminex is used to study serum levels of a panel of markers (e.g., CRP, IL-6, adrenalin and noradrenalin).

For the infiltration of immune cells in tumor, immunohistochemistry will be used to analyze biopsy material for parameters such as NK cells, T cells, B cells, MDSC, tumor-associated macrophages (TAMs) and dendritic cells (DC) and PD-L1 expression on tumor cells. Finally, single cell sequencing of immune cell subsets, in particular NK cells and T cells, will allow detection of exercise mobilized cell that have migrated to the tumor. Overall, the format of RCT allows all analysis to be compared to a reference samples from the control group. Finally, data will be correlated with clinical characteristics (VO_2_ max, ORR, PFS, OS and questionnaires).

### Statistical analysis

Primary outcome is exercise-induced mobilization of NK cells among cancer patients. To be able to detect an average difference in NK cell count between patients in the intervention group and patients in the control group of 50%, with an estimated standard deviation of 40, and to obtain a type I error rate of 5% and a power of 80%, a sample size of 17 patients per study arm will be needed. To account for an expected dropout rate of approximately 50%, we decided to increase this number to a group size of 35. Hence, a total of 70 patients will be included in the study.

All randomized patients will be assessable for safety assessment. Response rates are defined as the sum of the percentage of patients who achieved complete and partial response in each group divided by the number of patients. PFS is defined as the time interval between the date of randomization and the date of disease progression or the date of death of any cause. Patients will be censored at the time of last clinical contact if they are lost to follow-up or do not experience disease progression or die before the cut-off date for the analyses. OS will be calculated from the date of randomization to the date of death of NSCLC and any cause or last contact (censored observation).

Overall response rates (ORR) will be compared between the treatment groups using the χ2 test. Other categoric data will be compared using Fisher΄s exact test. The Kaplan –Meier method is used to estimate the distribution of time to events (i.e., PFS and OS).

### Status of trial

HI AIM first patients were included in August 2020. Due to the COVID-19 pandemic, the trial has been closed for several month, in order to limit the pressure on the Danish hospital system. As of January 2022, a total of 23 patients has been included in the trial.

## Discussion

NK and T cells of the immune system can recognize and kill cancer cells, and these cell types are found in greater or lesser numbers in tumors. In many different cancers, the presence of “many” immune cells—especially T cells—has been shown to be correlated with improved OS [40]. Thus, the immune system can spontaneously recognize and fight cancer cells and this ability has been exploited clinically, e.g. by administration of CPI [6]. Several characteristics of the patient can affect the response to treatment, but one of the strongest indicators of response is whether the tumor is “warm”, i.e., is infiltrated by immune effector cells [41, 42]. Furthermore, there are data to suggest that response to chemotherapy is also dependent on a functional immune system in the host [43]. Therefore, it may be of great importance to uncover methods of converting a "cold" non-infiltrated tumor into a "warm" immune cell infiltrated tumor. Thereby non-responding patients may be converted to "responders". Moreover, even responding patients may experience “deeper” responses and thus increase in the proportion of patients that obtain curative responses [44]. As a consequence, great efforts are being made to study the molecular and cellular background of lack of immune infiltration [41, 45], and ways by which immune cell infiltration can be increased [46]. We and others have recently shown that voluntary exercise has a remarkable effect on tumor growth in several mouse models [32, 47]. With HI AIM we take the first step, aiming to strengthen the notion that exercise is not only “healthy” but in connection with immunotherapy is also “therapeutic” [48]. To this end, demonstrating that immune cells can be mobilized in exercising LC patients, in turn leading to infiltration into the tumor, would set the stage for initiation of a larger trial focused on clinical efficacy.

In the study design, we have decided to focus on high intense aerobic exercise training, due to immune cell mobilization data shown in both man and mouse [49, 50]. Thus, it has been shown that immune cells egress to the peripheral blood upon a single bout of exercise [51], but also that the mobilization of T and NK cells are intensity dependent [52]. Therefore, aiming a high level of immune cell mobilization, high intensity would be desired. Quist et al. has previously shown that it is possible to conduct exercise training for patients with advanced NSCLC (stage IIIb-IV), but also that the patients actually improve on their physical capacity, anxiety level, and emotional well-being [53]. The study included 216 patients with NSCLC or SCLC, and consisted of a six-week supervised, and group-based exercise program consisting of aerobic exercise, strength training and relaxation. The aerobic exercise intensity was 85% to 95% of each patient’s maximum heart rate and lasted approximated 10–15 min. It was shown that high intensity aerobic exercise was feasible, even in a challenging patient group such as LC patients.

Only few prior studies have investigated exercise-induced mobilization of NK cells in cancer patients. In a pilot study from 2015, Evans et al*.* [54] investigated the impact of intermittent exercise (10 × three minutes interval training on a cycle ergometer at 60% of VO_2_ peak) among 9 sedentary healthy women and 9 women with stage I-III invasive breast cancer who had completed their planned chemotherapy and/or surgery or radiation therapy 3–6 months before enrollment. After 30 min of interval training the NK cell count significantly increased from 70.3 cells/µL (mean), SD ± 37.9 to 172.8 ± 118.8 among the breast cancer survivors. Both the baseline level and increase in NK after the exercise session were significantly lower compared to what was seen among the healthy sedentary women [54]. As these cancer survivors were in an early cancer stage and several months past treatment, we expect that the included LC patients in the current study, will have a more suppressed immune system and thus a more limited NK response to exercise.

Another important aspect of cell trafficking upon exercise, was shown by Rooney et al. [51], demonstrating that immune cells (including T and NK cells) extravasate the peripheral blood within 3 min of passive recovery. This underscore the importance of drawing blood directly after ended exercise session, in order to measure the exercise mediated mobilization of immune cells. Hence, for proper monitoring in the HI AIM trial, we have done several optimization steps and our data confirm that blood samples should be taken within 2 min, since a rapid decline in mobilized immune cells can be seen within only five and ten minutes after ended exercise (unpublished data). Overall, data from HI AIM will show if exercise lead to elevated numbers of immune effector cells (NK and T) in the peripheral blood and tumor, and hence, potentially be a key player in optimizing response to immunotherapy for LC patients.

A key question is related to whether exercise mobilized immune cells infiltrate the tumor. To this end, we have permission to take ultra-sound guided biopsies before and after exercise in patients that presents with accessible tumors. This will allow us to directly compare immune cell infiltration before and after exercise and for T cells to track individual exercise mobilized T cell clonotypes from peripheral blood to the tumor site. Moreover, taking advantage of multispectral fluorescence imaging it will be possible to get insight into the expression of markers for cytotoxic capacity of immune cells in tumors.

Concerning the efficacy of CPI-exercise therapy this will surely require bigger trials. However, data from HI AIM are geared to be informative regarding immune cell mobilization, and tumor infiltration of mobilized immune cells. Provided positive results, this will encourage the initiation of future trials that are scaled to study efficacy.

## Data Availability

Plans for data entry, security and storage have been approved by the Danish Data Protection Agency (P-2019–278). Datasets used and/or analysed during the current study are available from the corresponding author on reasonable request.

## Abbreviations

CCI: Charlson comorbidity index
CPI: Checkpoint inhibitors
CTLA-4: Cytotoxic T lymphocyte associated protein 4
HADS: Hospital Anxiety and Depression Scale
HI AIM: High Intensity Aerobic exercise training and Immune cell Mobilization in patients with lung cancer
HR_peak_: Heart rate peak
IPAQ: International Physical Activity Questionnaire
LC: Lung cancer
MWL: Maximal workload
NK cells: Natural killer cells
NSCLC: Non-small cell lung cancer
PD-1: Programmed cell death protein 1
PFS: Progression free survival
OS: Overall survival
QNR: Questionnaires
QoL: Quality of life
RCT: Randomized controlled trials
SCLC: Small cell lung cancer
TME: Tumor microenvironment
